# The effect of zinc supplementation in pre-diabetes: A protocol for systematic review and meta-analysis

**DOI:** 10.1097/MD.0000000000016259

**Published:** 2019-07-05

**Authors:** Xuqin Du, Lipeng Shi, Hong Gao, Xiaoxu Fu, Xiyu Zhang, Yuan Zhang, Chunguang Xie

**Affiliations:** a Teaching Hospital, School of Clinical Medicine, Chengdu University of Traditional Chinese Medicine, Chengdu, Sichuan Province; b Clinical Department, Dianjiang Hospital of Traditional Chinese Medicine, Dianjiang, Chongqing, China.

**Keywords:** meta-analysis, pre-diabetes, protocol, systematic review, zinc supplementation

## Abstract

**Background:**

The number of people with diabetes and pre-diabetes is growing exponentially. Human studies have shown that zinc supplementation is beneficial for pre-diabetes. However, owing to the low quality, small sample size, and methodological heterogeneity of these studies, this conclusion is not convincing. Consequently, in order to determine whether zinc supplementation is effective and safe in pre-diabetic patients, it is necessary to conduct a meta-analysis of high-quality clinical trials.

**Methods:**

We will retrieve MEDLINE (PubMed), EMBASE, the Web of Science, Cochrane Library, and the ClinicalTrials.gov website without restriction on language. Randomized controlled trials (RCTs) of Zinc supplementation for adult patients with pre-diabetes will be searched in multiple databases from inception to October 2020. The primary outcome of the meta-analysis is the HbA1c. The secondary outcomes include the fasting blood glucose (FBG), fasting insulin (FINS), homeostasis model assessment of insulin resistance (HOMA-IR), and quantitative insulin sensitivity check index (QUICKI). Two assessors will utilize the Cochrane Collaboration's risk of bias tool to evaluate the RCTs and all statistical data will be analyzed by using the Review Manage software V5.3.0.

**Results:**

This study will provide high-quality synthesis of effectiveness and safety of zinc supplementation for pre-diabetes.

**Conclusion:**

This systematic review and meta-analysis will provide the available evidence to assess whether the zinc supplementation is beneficial to glucose control and insulin resistance in patients with pre-diabetes.

**PROSPERO registration number:**

CRD 42018095724

## 1 Introduction

Pre-diabetes, also known as impaired glucose regulation, refers to the intermediate metabolic state between normal glucose homeostasis and diabetic hyperglycemia, including impaired fasting glucose regulation and impaired glucose tolerance.^[1]^ The number of people living with diabetes and pre-diabetes is exponentially growing worldwide, on account of population growth, aging, urbanization, unhealthy eating habits, lack of physical activity, and rising prevalence of obesity.^[2]^ Based on the analysis of relevant data from 110 countries, the IDF predicts that the number of global diabetes patients will reach 592 million in 2035.^[3]^ In China, the prevalence of type 2 diabetes and pre-diabetes above 18 years old is as high as 11.6% and 50.1%, respectively.^[4]^ It is reported that 70% of pre-diabetic patients will develop diabetes at different times.^[5]^ Diabetes has led to a heavy medical burden and a large indirect social cost, with an annual global investment of more than $827 billion.^[6]^ Reduced productivity due to restrictions on daily activities also imposes a heavier burden. Consequently, it is timely to take simple and effective preventive measures for pre-diabetic patients.

Ninety percent of diabetic patients are type 2 diabetes, characterized by insulin resistance and β-cell dysfunction.^[7]^ Insulin is mainly stored in pancreatic β-cells in the form of a dizinc insulin hexamer. Studies have shown that zinc is closely related to the synthesis, secretion, storage, degradation, and biological activity of insulin.^[8,9]^ Zinc can improve the stability and bioavailability of insulin.^[10,11]^ Meanwhile, zinc has an insulin-like effect,^[12]^ which increases the activity of the insulin signaling pathway and exerts an anti-diabetic effect.^[13]^ Insulin degrading enzymes, as zinc-containing enzymes, are the most important enzymes for catalyzing insulin degradation at the cellular level. In vivo data suggests that zinc-deficient mice have a reduced ability to metabolize glucose in high insulin-normal blood glucose clamp experiments.^[14]^ Zinc is momentous in carbohydrate and protein metabolism.^[8,9]^ Simultaneously, oxidative stress and inflammation are important pathogenesis of diabetes mellitus and its complications. Zinc deficiency leads to increased oxidative stress, activation of mononuclear macrophages, and increased production of inflammatory factors.^[15]^ Studies have indicated that diabetes mellitus is accompanied by hypozincemia.^[16,17]^ In addition, zinc deficiency is more prevalent in developing countries,^[18]^ where the prevalence of diabetes and pre-diabetes is also increasing exponentially.^[2]^

Human studies and animal experiments have demonstrated that zinc supplementation improves glucose and lipid metabolism, and that there is an enhanced ability of anti-oxidative stress in diabetes.^[19–21]^ In vivo studies in humans have also shown that zinc supplementation is beneficial for pre-diabetes.^[22,23]^ However, systematic reviews are generally more competent and less biased than the individual studies included, and the careful collection of therapeutic effects can provide the most accurate overall assessment of interventions.^[24]^ There are currently no systematic reviews to explore the therapeutic effect of zinc supplementation in pre-diabetic humans. The aim of this study is to systematically evaluate the literature and meta-analyze the therapeutic effects of zinc supplementation in pre-diabetic humans and to evaluate the potential toxic effects of zinc supplementation in the literature.

## 2 Methods

### 2.1 PROSPERO registration

This protocol has been registered on PROSPERO with registration number PROSPERO CRD42018095724. (http://www.crd.york.ac.uk/PROSPERO/display_record.php?ID=CRD42018095724).

### 2.2 Eligibility criteria

#### 2.2.1 Types of trials

Only RCTs of Zinc supplementation for humans with pre-diabetes will be included in this study. We will exclude observational, cohort, case-control, case series, and laboratory studies.

#### 2.2.2 Types of patients

Patients with pre-diabetes (aged ≥ 18 years) will be included.

#### 2.2.3 Types of interventions

This meta-analysis will include the RCTs of zinc supplementation regardless of dose and frequency. Trials with a minimum treatment duration of at least 4 weeks will be included.

#### 2.2.4 Types of controls

This meta-analysis will include the RCTs that administered with placebo, that with no treatment or active pharmacological treatment (regardless of dose, frequency). Trials with a minimum treatment duration of at least 4 weeks will be included.

#### 2.2.5 Types of outcome measurements

##### 2.2.5.1 Primary outcomes

The primary outcome of this review is HbA1c.

##### 2.2.5.2 Secondary outcomes

The secondary outcomes include the following items: fasting blood glucose (FBG), fasting insulin (FINS), homeostasis model assessment of insulin resistance (HOMA-IR), quantitative insulin sensitivity check index (QUICKI).

### 2.3 Search methods for the identification of eligible trials

We will conduct a systematic literature retrieval of relevant databases including MEDLINE, EMBASE, the Web of Science, Cochrane Library, and the ClinicalTrials.gov website from inception to October 2020 without language restrictions. The following search terms will be used in various combinations and applicable to each database: pre-diabetes, prediabetes, impaired fasting glucose, impaired glucose tolerance, glucose intolerance, zinc, randomized controlled trial, randomised controlled trial, controlled clinical trial, clinical trial, randomized, randomised, trial. The search strategy on PubMed MEDLINE is listed in Table 1.

**Table 1:**
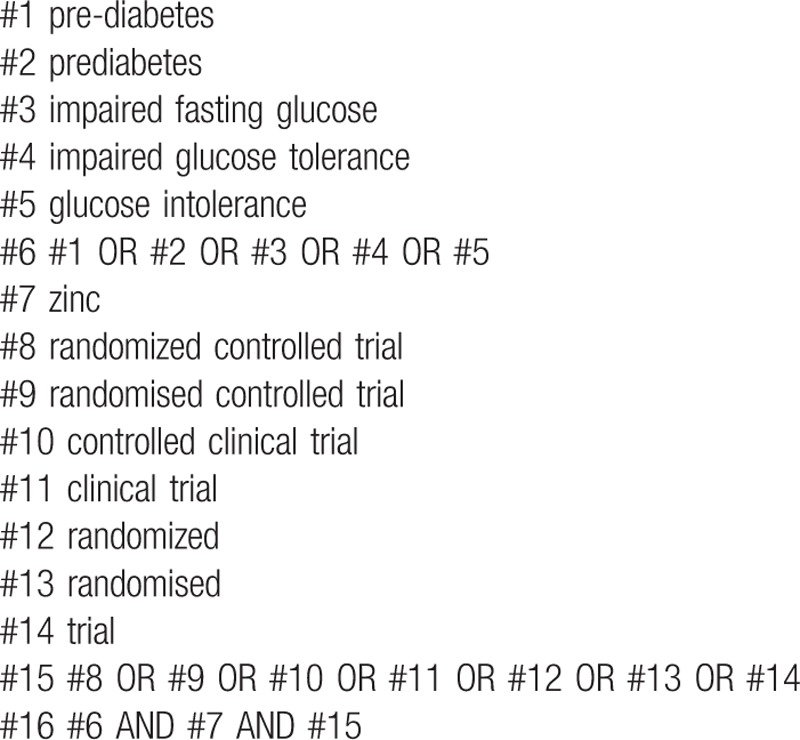
PubMed Medline search strategy.

### 2.4 Study selection

**Figure 1:**
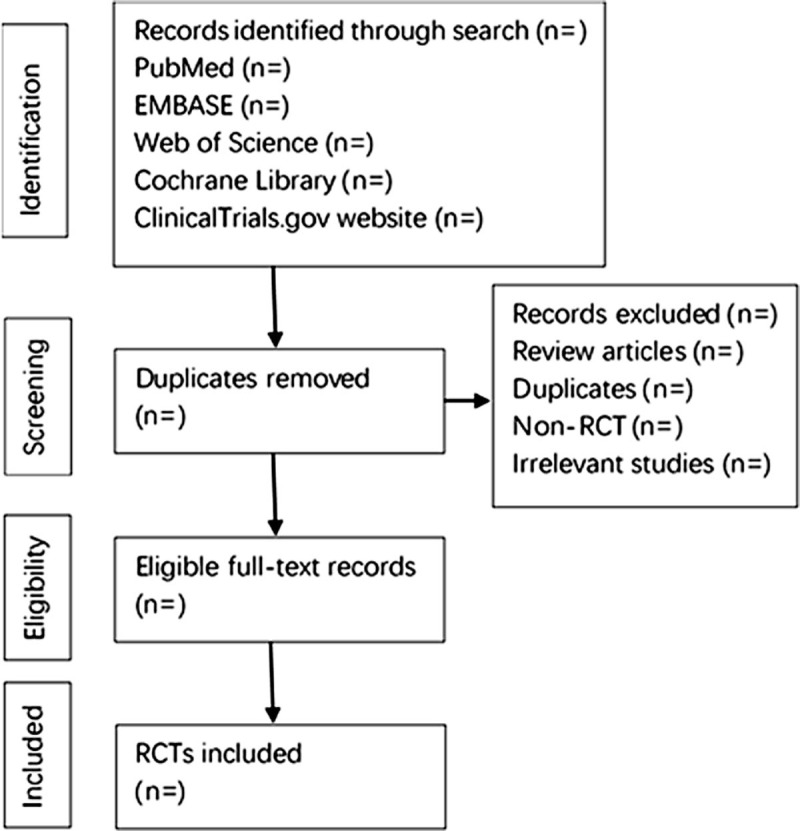
Flow chart of the study selection.

Two reviewers will independently retrieve all the literature. The references identified from relevant database searches will be imported into the EndNote software. After that, duplicate records will be removed. The reviewers first screen the title and abstract of each citation to identify potentially eligible studies and then review the full text to confirm inclusion. Any disagreement will be settled through consultation or negotiation with a third reviewer. Our findings will be reported using the recommended methods and checklist of the Preferred Reporting Items for Systematic Reviews and Meta-Analyses (PRISMA).^[25]^ We created this protocol using the PRISMA Protocols guidelines.^[26]^ In addition, the flow chart (Fig. 1) will be applied to describe the identification and selection process of the study.

### 2.5 Data extraction and management

Based on the eligibility criteria, 2 reviewers will evaluate the studies by using the same eligibility evaluation form. The following information will be documented from all the included studies: study characteristics (title, first author, publication year, study design, sample size, setting, randomization methodology, allocation concealment, blinding), participant characteristics (age, gender, number in each group, etc.), intervention details (type of interventions, type of controls, dose, route of administration, duration of treatment or follow-up, number of cases included in the statistical analysis, etc.), outcome indicators for efficacy and safety. Any disagreements will be rechecked and discussed. If no agreement can be achieved, the final decision will be consulted with a third reviewer.

### 2.6 Missing data management

The missing relevant data will not be included in the analysis if they are still not available after contacting the author. To investigate whether these missing data will affect the results of the meta-analysis, we will conduct a sensitivity analysis.

### 2.7 Risk of bias assessment

Two review authors will independently evaluate the design, execution, and reporting of the included RCTs based on the Cochrane risk of bias tool. The following 7 items affiliated with bias risk, including random sequence generation, allocation concealment, blinding of participants and personnel, blinding of outcome assessment, incomplete outcome data, selective outcome reporting, and other biases, will be evaluated by 2 reviewers. In addition, the risk for each item will be graded as high, low, or unclear. The disagreement of bias risk will be resolved by further discussion or consultation to a third independent reviewer.

### 2.8 Statistical analysis

The risk ratio (RR) for dichotomous data will be calculated, respectively, along with 95% CI. For continuous data, the mean difference (MD) or standardized mean difference (SMD) with 95% CI will be estimated. If the same scale is employed across different studies to measure an outcome, we will utilize the MD. Also, if different scales are employed to measure the same outcome, we will utilize the SMD. If an outcome measure includes less than 2 trials, we will descriptively summarize the results.

Statistical heterogeneity among studies will be evaluated using the Cochran Q test (χ2) and the I^2^ statistical value. We will categorize the heterogeneity using the following rules. I^2^ of 0% to 25% suggests low heterogeneity. I^2^ of 25% to 50% represents moderate heterogeneity. And I^2^ of 75% to 100% represents high heterogeneity. When the *P* value from a χ2 test is more than .10 or I^2^ ≤50%, we will adopt the fixed-effects model. Otherwise, there will be detectable variations between studies. Subgroup analysis will be performed to identify possible explanations for statistical heterogeneity, taking into account prespecified factors.

We will utilize the Review Manage software V5.3.0 (The Nordic Cochrane Center, The Cochrane Collaboration, 2014, Copenhagen, Denmark) to statistically analyze all data. The overall RR with its 95% CI for dichotomous data will be estimated. The MD or SMD with 95% CI will be calculated for continuous data in different situations. The fixed-effects model will be employed as appropriate for analysis. If the heterogeneity in the study is considerable, subgroup analysis will be conducted to investigate possible sources of statistical heterogeneity. When a meta-analysis is not available, descriptive summaries of individual findings will be provided.

### 2.9 Additional analysis

#### 2.9.1 Subgroup analysis

We will analyze subgroups based on different dosages of zinc and duration of treatment.

#### 2.9.2 Sensitivity analysis

To identify the robustness of the meta-analysis results, we will conduct a sensitivity analysis by omitting each of the RCT, or excluding the RCTs with high risk of bias, or excluding the RCTs with missing data.

#### 2.9.3 Reporting bias

If there are more than 10 studies in the meta-analysis, the symmetry of the funnel plot will be assessed to examine publication bias, with results being interpreted cautiously.

#### 2.9.4 Confidence in cumulative evidence

Two reviewers will independently evaluate the quality of the evidence for each outcome by using the Grading of Recommendations Assessment, Development and Evaluation (GRADE) system. According to the 5 factors (limitation, inaccuracy, inconsistency, indirectness, and publication bias) that could reduce the quality of evidence in the scoring system, the quality of evidence will be divided into 4 grades: high, moderate, low, and very low. The GRADE profiler 3.2 will be employed for analysis.

## 3 Discussion

A meta-analysis of high-quality trials will provide the most reliable evidence for the clinical treatment of pre-diabetes. The purpose of this systemic review and meta-analysis is to evaluate the effectiveness and safety of zinc supplementation in pre-diabetic humans. We will identify the influence of effectiveness and safety in different dosages of zinc and different duration of treatment. Overall, we will give a comprehensive picture of efficacy and adverse events in patients treated with the zine. In order to guarantee the accuracy and reliability of the results, the articles will be independently screened by different authors at least three times. Herein, this systemic review and meta-analysis will be the first to assess the effectiveness and safety of zinc supplementation in pre-diabetic humans, which may offer a comprehensive understanding of zinc supplementation in pre-diabetes.

## Author contributions

**Conceptualization:** Xuqin Du, Lipeng Shi, Hong Gao.

**Data curation:** Xiaoxu Fu.

**Funding acquisition:** Hong Gao.

**Investigation:** Lipeng Shi.

**Methodology:** Xuqin Du, Lipeng Shi.

**Project administration:** Xuqin Du, Chunguang Xie.

**Software:** Yuan Zhang.

**Supervision:** Xiyu Zhang.

**Validation:** Chunguang Xie.

**Writing – original draft:** Xuqin Du, Lipeng Shi.

**Writing – review & editing:** Xuqin Du, Lipeng Shi.

Xuqin Du orcid: 0000-0002-6782-4122.
